# PARP1 bound to XRCC2 promotes tumor progression in colorectal cancer

**DOI:** 10.1007/s12672-024-01112-y

**Published:** 2024-06-21

**Authors:** Kaiwu Xu, Zhige Yu, Tailiang Lu, Wei Peng, Yongqiang Gong, Chaowu Chen

**Affiliations:** Department of Gastrointestinal Surgery, Hunan Provincial People’s Hospital, The First Affiliated Hospital of Hunan Normal University, Changsha, 410005 People’s Republic of China

**Keywords:** PARP1, XRCC2, Progression, Colorectal cancer

## Abstract

**Background:**

By complexing poly (ADP-ribose) (PAR) in reaction to broke strand, PAR polymerase1 (PARP1) acts as the key enzyme participated in DNA repair. However, recent studies suggest that unrepaired DNA breaks results in persistent PARP1 activation, which leads to a progressively reduce in hexokinase1 (HK1) activity and cell death. PARP-1 is TCF-4/β-A novel co activator of gene transactivation induced by catenin may play a role in the development of colorectal cancer. The molecular mechanism of PARP1 remains elusive.

**Methods:**

212 colorectal cancer (CRC) patients who had the operation at our hospital were recruited. PARP1 expression was evaluated by immunohistochemistry. Stable CRC cell lines with low or high PARP1 expression were constructed. Survival analysis was computed based on PARP1 expression. The cell proliferation was tested by CCK-8 and Colony formation assay. The interaction of PARP1 and XRCC2 was detected by immunoprecipitation (IP) analysis.

**Results:**

Compared with matching adjacent noncancerous tissue, PARP1 was upregulated in CRC tissue which was correlated with the degree of differentiation, TNM stage, depth of invasion, metastasis, and survival. In addition, after constructing CRC stable cell lines with abnormal expression of PARP1, we found that overexpression of PARP1 promoted proliferation, and demonstrated the interaction between PARP1 and XRCC2 in CRC cells through immunoprecipitation (IP) analysis. Moreover, the inhibitor of XRCC2 can suppress the in vitro proliferation arousing by upregulation of PARP1.

**Conclusions:**

PARP1 was upregulated in CRC cells and promoted cell proliferation. Furthermore, the expression status of PARP1 was significantly correlated with some clinicopathological features and 5-year survival.

## Introduction

Colorectal cancer (CRC), which ranks fourth (6.1%) in the light of incidence but second (9.2%) in accordance with mortality in diagnosed cancer, takes a place of the most usual gastrointestinal cancers worldwide. Currently, in 2018, around 1.8 million people were confirmed CRC worldwide, and over 881,000 patients died of the disease, occupying about 10% cancer cases and deaths [1]. More importantly, in recent decades, the CRC incidence and mortality have increased in China [2]. Scholars have made progress in targeted therapy of CRC; nevertheless, superior targeting drugs are desired since the current treatment cannot generate satisfactory results.

Changes in PARP1 levels play a vital role in CRC [3]. PARP1 plays a fundamental role in preserving genome stability and regulating chromatin structure. PARP1 takes part in several DNA repair pathways, which include DNA single-strand break (SSB) repair, HR repair (HRR) pathway of DNA double-stranded breaks (DSBs), and base excision repair (BER). HRR-deficient neoplasms are extremely susceptive to PARP1 inhibitors based on the synthetic lethality theory [4–6]. PARP1 inhibitors mainly prevent the PARP1 catalytic activity and continuously increase the single-strand breaks levels, resulting in DNA DSBs upon replication [5, 7, 8]. On the other hand, unrepaired DNA breaks, arising from DNA repair deficiency and/or overexposure of genotoxin, results in persistently PARP1 activated and cell death [9]. Uncontrolled or excessive activation of PARP1 leads to numerous pathological results, including the onset of diabetes, streptozotocin-induced pancreatic beta-cell death, myocardial ischemia, and tissue injury from cerebral [10–13]. What’s more, the study of Fouquerel [14] has demonstrated that by inhibiting HK1 independent of NAD+ depletion, PARP1 regulates glycolysis negatively. The result showed that activated PARP1 inhibited the activity of HK1, thereby inhibiting ATP synthesis and cell death.

Therefore, PARP1 as an important member of DNA damage repair and energy metabolism pathway of tumor cells, its specific role and mechanism have not been clarified. In this research, we intend to study the effect of PARP1 in CRC cells and provide an important basis for PARP1 to become a new therapeutic target for CRC.

## Materials and methods

### Patients

From October 2010 to December 2012, 212 primary CRC tissues and 47 matching adjacent noncancerous tissues were obtained from patients who had the operation at the Hunan Provincial People’s Hospital. Patients with preoperative chemotherapy and/or radiotherapy, and palliative surgery were excluded. Survival analysis excluded patients who died within 30 days after surgery, since their death could be attributed to surgical complications. All patients have signed an informed consent. The Ethical Review Board of Hunan Provincial People’s Hospital have approved the study. The study complied with the guidelines of the Declaration of Helsinki.

### Immunohistochemistry (IHC)

According to previously described methods, we performed the following procedures by the classic biotin–streptavidin–peroxidase IHC staining protocols [15]. We obtained sections from the Hunan Provincial People’s Hospital Pathology Department, and incubated overnight at 48 °C with polyclonal primary antibody against PARP1 (1:100; Abcam, Cambridge, UK). Following incubated with diaminobenzidine and horseradish peroxidase-conjugated sheep anti-rabbit secondary antibody (Beyotime; Guangzhou, China), used Mayer’s hematoxylin to counterstain the slides. Positive control was primary CRC tissue section. In negative control staining, used phosphate-buffered saline (PBS) buffer instead of primary antibody. The immunostaining results were scored according to the methods previously described below [16].

### Culture and treatment of cell

We obtained human CRC cell lines FHC, SW480, SW620, LoVo, SW403, HT-29, COLO205 and COLO320DM from the American Type Culture Collection (Manassas, VA, USA). Incubated cell lines in DMEM/RPMI-1640 medium (Gibco, Thermo Fisher Scientific, Waltham, MA, USA) supplemented with penicillin (100 U/mL), streptomycin (100 μg/mL), and 10% FBS (Gibco, Thermo Fisher Scientific, Waltham, MA, USA) at 37 °C with 5% CO_2_.

### Vectors, retroviral infection and transfection

Through subcloning the PCR-amplified human PARP1 coding sequence into a pBABE-puro vector, we generated pBABE/PARP1-overexpressing human PARP1. Cloned two RNA interference (RNAi) oligonucleotides into pSuper-retro-puro vectors to produce the pSuper-retro-PARP1-RNAi respectively, thereby silencing endogenous PARP1. As mentioned earlier, the generation and infection of retroviruses were carried out to establish stable cell lines [17]. The calcium phosphate transfection method was used to cotransfect the retroviruses into 293FT cells and then harvested and infected cells. After infection 48 h, cell line stably overexpress of PARP1 and the vector control name as (SW480/PARP1; SW480/vector, SW620/PARP1, SW620/vector, respectively) or PARP1 RNAi and the Negative control (SW480/PARP1/RNAi, SW480/Scramble, SW620/PARP1/RNAi; SW620/Scramble, respectively) were selected using puromycin (0.5 mg/mL) over 10 days. SDS-PAGE was used to segregate SW480 and SW620 cell lysates to detect PARP1 protein levels, and the cell proliferation was analyzed with the stable cell line. The XRCC2 inhibitor (XRCC2/siRNA), negative control (NC) were purchased from RiboBio Co. Ltd (Guangzhou, Guangdong, China). Transfection of oligonucleotides was performed using the Lipofectamine 2000 reagent (Invitrogen, Carlsbad, CA), according to the manufacturers’ protocol.

### Extraction and reverse transcription of RNA, real-time quantitative PCR

In line with manufacturer’s illustrations, applied Trizol reagent (Invitrogen, Carlsbad, CA, USA) to accomplish total RNA extraction from cultured cells or tissues. cDNAs were amplified and equipped with the ABI PRISM 7500 system (Applied Biosystems, Foster City, CA, USA), SYBR Green I (Invitrogen, Carlsbad, CA, USA) was applied for real-time quantitative PCR. Selected housekeeping gene GAPDH as an internal control. Used the primers below: PARP1 forward, 5′-ACAGTGTGCAGGCCAAGGTG-3′, and reverse 5′-CTCGGC TTCTTCAGAATCTCTGTC-3′; XRCC2 forward: 5′-TCACCTGTGCATGGTG ATATT-3′, and reverse: 5′-TTCCAGGCCACCTTCTGATT-3′; GAPDH forward: 5′-GACTCATGACCACAGTCCATGC-3′, and reverse: 5′-AGAGGCAGGGATGATG TTCTG-3′; p21 forward: 5′-CGATGCCAACCTCCTCAACGA-3′, and reverse: 5′-TCGCAGACCTCCAGCATCCA-3′; cyclin D1 forward: 5′-AACTACCTGGA CCGCTTCCT-3′, and reverse: 5′-CCACTTGAGCTTGTTCACCA-3′. Genes expression data were normalized to the geometric mean of housekeeping gene GAPDH to control the variability in expression levels and calculated as 2^−[(*Ct* of gene)^ ^−^ ^(*Ct* of GAPDH)]^, where Ct represents the threshold cycle for each transcript.

### Western blotting

According to manufacturer’s instructions, proteins were prepared from cell lysates, BCA method for determining protein content; isolated on SDS-PAGE, and transferred to PVDF membranes. PVDF membranes were cut prior to hybridisation with antibodies during blotting. To detect specific proteins, primary antibodies that were used included α-Tubulin mouse monoclonal antibody (1:1000; Sigma-Aldrich, St. Louis, MO, USA), anti-human XRCC2 mouse monoclonal antibody (1:1500; Abcam), anti-human PARP1 mouse monoclonal antibody (1:1500; Abcam), anti-human cyclin D1 rabbit monoclonal antibody (1:1500; Abcam), and anti-human P21 rabbit monoclonal antibody (1:1500; Abcam). The secondary antibody was goat anti-mouse antibody (1:2000; Santa Cruz Biotechnology, Santa Cruz, CA, USA). After the membrane incubated with Clarity enhanced chemiluminescence (ECL), and we cut the X-ray film to a suitable size for development. An anti-α-tubulin antibody was used as a loading control. At least three biological replicates were used to detect the sample.

### Cell proliferation detection

In accordance with the manufacturer’s illustrations, used the Cell Counting Kit-8 (CCK-8) cell proliferation kit (Dojindo Laboratories, Kumamoto, Japan) to assess cell proliferation. Concisely, seeded the cells into 96-well plates (2 × 103 cells/well), and cultured under regular circumstances with 100 μL complete medium. At the specified time, incubated cells with RPMI-1640 medium (100 μL) plus CCK8 reagent (10 μL) for 2 h at 37 °C. After that, measured the absorbance at 450 nm wavelength on a microplate reader (Bio-Rad, La Jolla, CA, USA). Conducted three repetition experiments independently.

### Colony formation assay

In brief, plated exponential growth cells into 6-well plates at 1000 cells/well and cultured for 10–14 days at 37 °C with 5% CO_2_. For visualization and counting, used 75% ethanol to fix the colonies for 30 min and stained with 0.5% crystal violet (Beyotime, Nanjing, China) afterwards. When colonies with more than 50 cells would be manually calculated. Every group of cells comprised three wells, and three independent repeat experiments were conducted.

### Co-immunoprecipitation (Co-IP)

Co-IP was carried out using Pierce Co-Immunoprecipitation Kit (cat. 26149, Thermo Fisher Scientific, Waltham, MA, USA) according to the protocol provided by the manufacturer. SW620 cells and anti-human XRCC2 mouse monoclonal antibody (1:1500; Abcam, Cambridge, MA, USA), anti-human PARP1 mouse monoclonal antibody (1:1500; Abcam, Cambridge, MA, USA) were used.

### Statistical analysis

Statistical analysis was conducted using SPSS 20.0 (SPSS Inc, Chicago, IL, USA). Employed the Chi-square test to evaluate the association between PARP1 expression and clinicopathological characteristics. The significant differences between two groups of data were analyzed with the Student’s t test. The log-rank test and the Kaplan–Meier method were employed for survival curves analysis. The time from surgery to last follow-up date or patient’s death was 5-year overall survival (OS). The time from curative surgery to recurrence, the final follow-up date, or death was defined as Relapse-free survival (RFS). Local and distant relapses were regarded as recurrence. The statistical significance was set at *p* < *0.05*.

## Results

### PARP1 was upregulated in CRC cell lines and associated with clinical prognosis

**Fig. 1:**
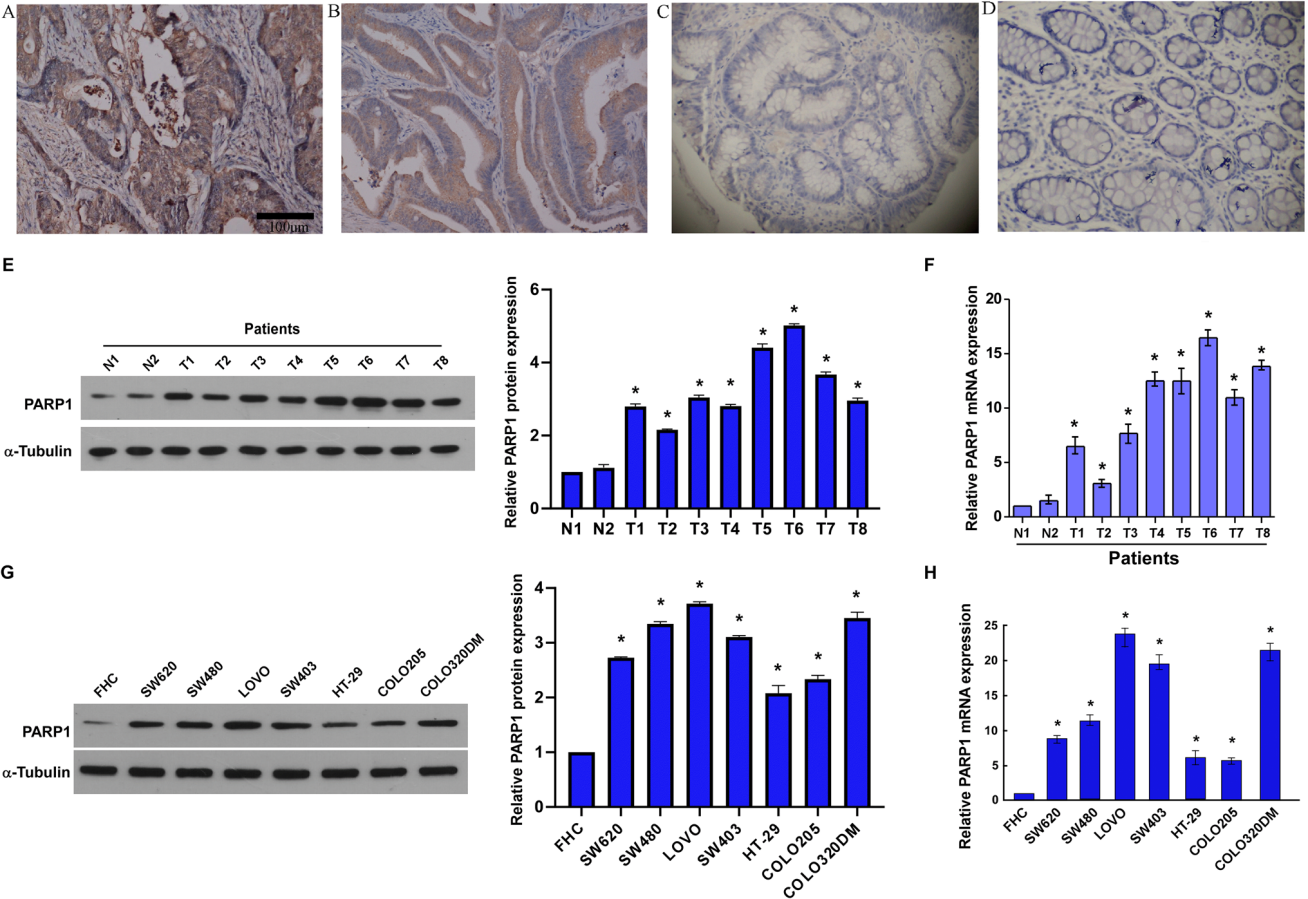
PARP1 expression in CRC and adjacent normal mucosal tissues. There was **A** positive and **B** weakly positive PARP1 expression in primary CRC (×200 magnification). There was negative PARP1 expression in primary CRC (**C**) and normal colorectal mucosa tissue (**D**). Western blotting (**E**) and real-time PCR (**F**). PARP1 expression was examined in eight CRC cell lines and in normal colonic epithelial cell (FHC) using Western blotting (**G**) and real-time PCR (**H**). (*p* < 0.05; Fig. 2C, D). Blot menbranes were cut prior to hybridisation with antibodies during blotting and developed with X-ray film. The scale bar represents 100 µm. *p < 0.05, (Student’s t test)

The expression of PARP1 in primary CRC tissue and matching adjacent noncancerous tissue was described in Fig. 1. In 157 of 212 (74.1%) primary CRC tissues, positive PARP1 staining was discovered. By contrast, compared with primary CRC tissue, in the matching adjacent noncancerous tissues, PARP1 staining positive rate was only 53.2% (25/47 samples, *p* = *0.005*; Fig. 1A–D; Table 1). Furthermore, applied western blotting and real-time PCR to detect eight CRC patients PARP1 expression in tumors and the matching adjacent noncancerous tissues. Different from normal tissues, PARP1 was upregulated remarkably in tumors (*p* < *0.05*; Fig. 1E, F). Moreover, we also used western blotting and real-time PCR to examine PARP1 expression in normal colonic epithelial cell (FHC) and eight CRC cell lines. Compared with colonic epithelial cell, PARP1 was upregulated remarkably in CRC cell lines (*p* < *0.05*; Fig. 1G, H).

**Table 1:** PARP1 expression in primary CRC tissue and adjacent noncancerous tissue

Tissue sample	n	Expression of PARP1		P
		Positive (%)	Negative (%)	
Primary colorectal cancer	212	157 (74.1)	55 (25.9)	0.005
Adjacent normal colorectal mucosa tissues	47	25 (53.2)	22 (46.8)	

### Correlation between PARP1 expression and CRC patients clinicopathological characteristics

Table 2 described the relevance between PARP1 expression and clinicopathological features, including age, gender, differentiation, distant metastasis, tumor-nodes-metastasis (TNM) stage, tumor size, lymph node metastasis, tumor site, and depth of invasion. The expression status of PARP1 in primary CRC was remarkably associated with the degree of differentiation, TNM stage, distant metastasis, and depth of invasion (*p* = *0.015*; *p* = *0.002*; *p* = *0.001*; *p* = *0.001*, respectively).

**Table 2:** Clinicopathological characteristics and PARP1 expression status of patients with colorectal cancer

Clinical characteristics	n	Positive (%)	Negative (%)	P
Gender				
Male	102	73 (71.6)	29 (28.4)	0.426
Female	110	84 (76.4)	26 (23.6)	
Age				
< 50y	55	44 (80.0)	11 (20.0)	0.503
50–70y	122	88 (72.1)	34 (27.9)	
> 70y	35	25 (71.4)	10 (28.6)	
Tumor site				
Rectum	83	60 (72.3)	23 (27.7)	0.83
Left	85	63 (74.1)	22 (25.9)	
Right	44	34 (77.3)	10 (22.7)	
Tumor size				
< 3 cm	24	17 (70.8)	7 (29.2)	0.149
3–5 cm	111	77 (69.4)	34 (30.6)	
> 5 cm	77	63 (81.8)	14 (18.2)	
Lymph node metastasis				
Yes	144	112 (77.8)	32 (22.2)	0.072
No	68	45 (66.2)	23 (33.8)	
Distant metastasis				
Yes	54	49 (90.7)	5 (9.3)	0.001*
No	158	108 (68.4)	50 (31.6)	
Depth of invasion				
T1	8	3 (37.5)	5 (62.5)	0.001*
T2	20	9 (45.0)	11 (55.0)	
T3	45	33 (73.3)	12 (26.7)	
T4	139	112 (80.6)	27 (19.4)	
Degree of differentiation				
High	4	1 (25.0)	3 (75.0)	0.015*
Moderately	161	116 (72.0)	45 (28.0)	
Low	47	40 (85.1)	7 (14.9)	
TNM				
I/II	37	20 (54.1)	17 (45.9)	0.002*
III/IV	175	137 (78.3)	38 (21.7)	

*p < *0.05*

### Survival analysis and prognostic significance of PARP1 expression

**Fig. 2:**
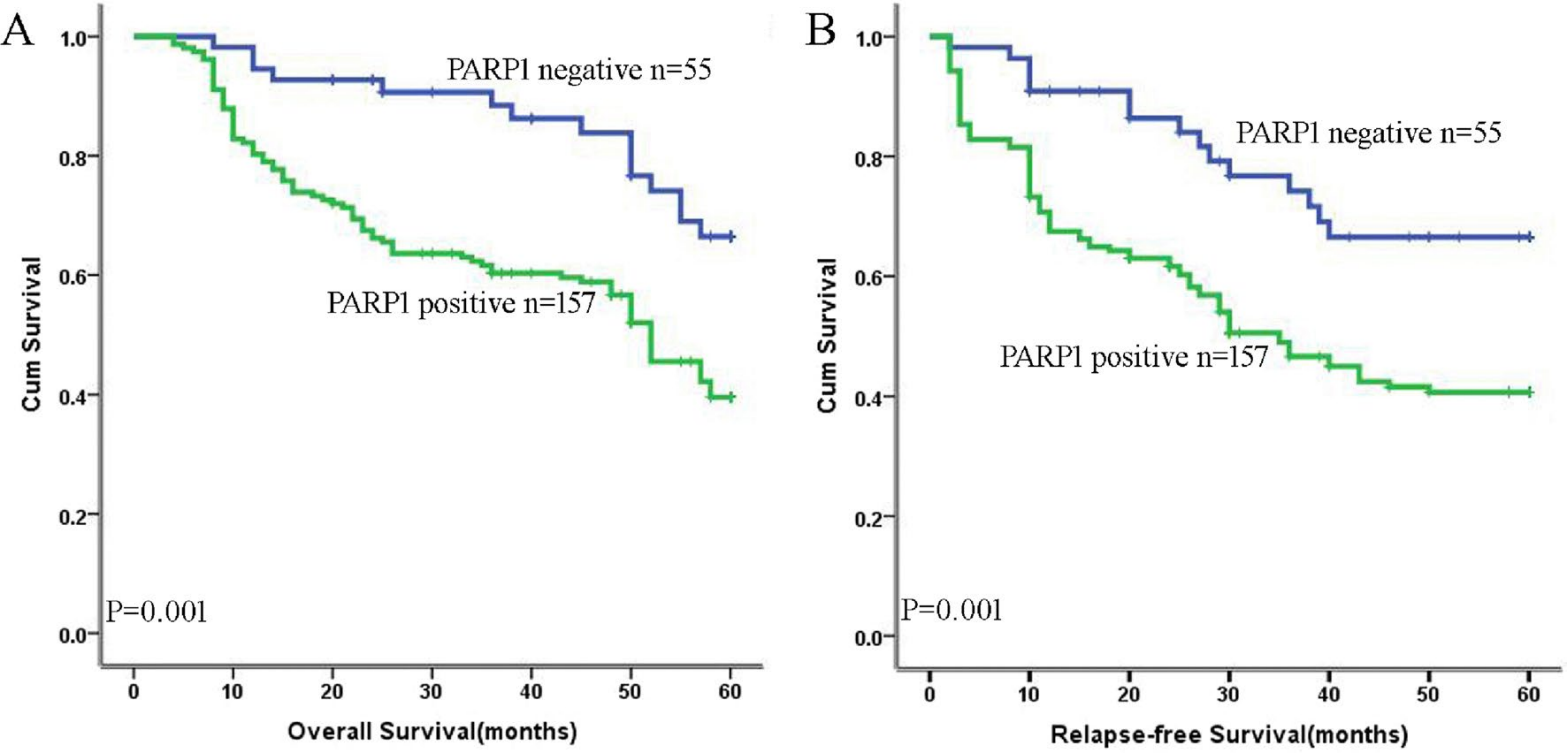
Survival curve in regards to PARP1 expression and the PARP1 expression in SW480/SW620 cells. Patients with positive PARP1 expression have shorter OS (**A**) and RFS (**B**) than patients with negative PARP1 expression (*p* = *0.001*; *p* = *0.001*, respectively)

Figure 2A, B showed the Kaplan–Meier estimates for the group with positive PARP1 and group with negative PARP1. The median time to total OS for 212 patients was 43.9 months. And the median OS time for two groups was 40.7 months and 53.1 months separately. The two survival curves were remarkably different (Fig. 2A; *χ*^*2*^ = *12.095*; *p* = *0.001*). 212 patients total median RFS time was 38.0 months. While the median RFS time for two groups was 34.7 months and 48.1 months respectively. The two survival curves were also significantly different (Fig. 2B; *χ*^*2*^ = *10.848*; *p* = *0.001*). These results showed that, compared with negative PARP1 expression, patients with positive PARP1 expression had shorter OS and RFS.

### Stable cell lines expressing high or low PARP1

**Fig. 3:**
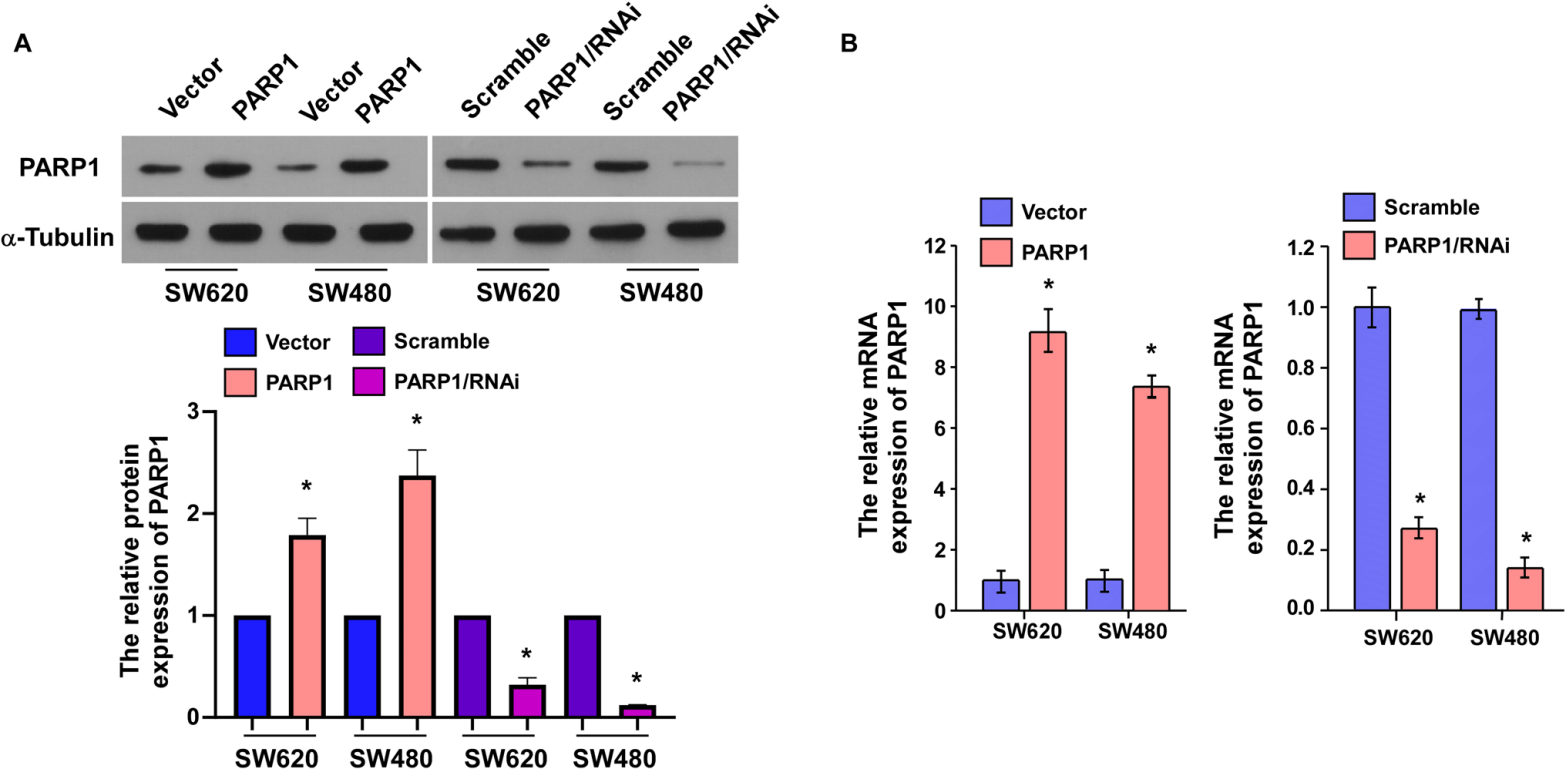
**A** Western blotting analysis of PARP1 expression in SW480/SW620-Vector, SW480/SW620-PARP1, SW480/SW620-Scramble, and SW480/SW620-PARP1/RNAi cells. **B** QPCR analysis of PARP1 mRNA expression in SW480 and SW620 cells. Blot menbranes were cut prior to hybridisation with antibodies during blotting and developed with X-ray film.*P < *0.05*, (Student’s t test)

Western blotting and quantitative PCR (QPCR) was used to detect PARP1 expression after cell lines expressing high or low PARP1 were constructed. Western blotting revealed that PARP1 expression in SW480/SW620-PARP1/RNAi was lower than that in SW480/SW620-Scramble cells. What’s more, PARP1 expression in SW480/SW620-PARP1 cells was higher than that in SW480/SW620-Vector cells (Fig. 3B; *p* < *0.05*). Moreover, the results obtained by QPCR were similar (Fig. 3A; *p* < *0.05*). The above results indicated that stable cell lines expressing low or high PARP1 had been successfully constructed.

### PARP1 overexpression promoted proliferation in CRC cells

**Fig. 4:**
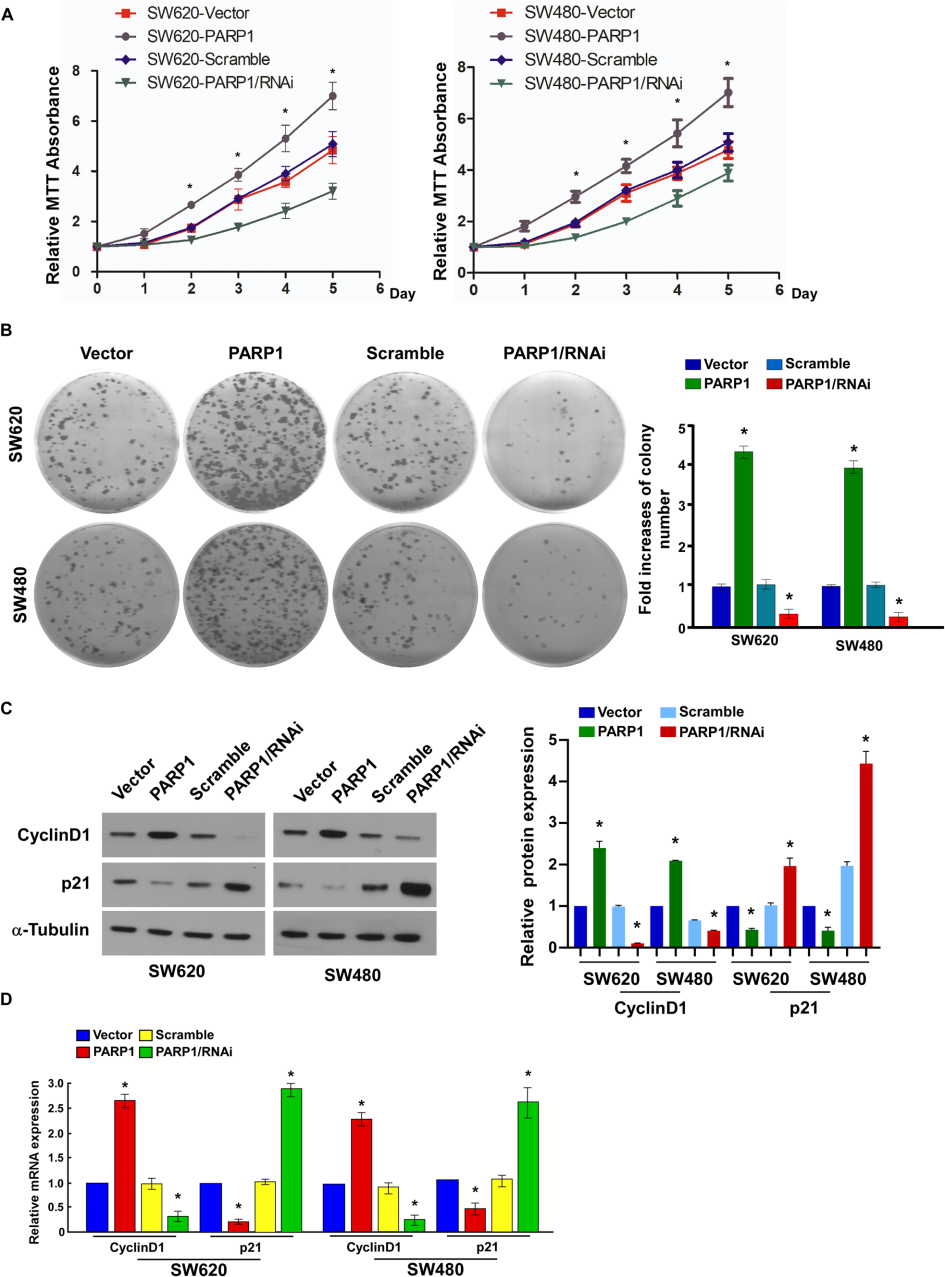
Cell proliferation analysis. CCK-8 assay (**A**) and Colony formation assay (**B**) of SW480/SW620 cells transfected with PARP1, PARP1/RNAi, and Vector/Scramble. **C** Western blot analysis of cyclin D1 and p21 expression. **D** QPCR analysis of cyclin D1 and p21 expression. Blot membranes were cut prior to hybridisation with antibodies during blotting and developed with X-ray film. *p < *0.05*, (Student’s t test)

Through CCK-8 assay and colony formation assay, we assessed PARP1 overexpression effect on CRC cell proliferation to study whether PARP1 affects CRC development and progression. Compared with PARP1/RNAi, PARP1 upregulation remarkably increased SW480/SW620 cells growth rate 48 h after PARP1 transduction **(**Fig. 4A, B; *p* < *0.05*). As expected, PARP1 overexpression increased the expression of cyclin D1, while significantly reducing p21 expression in CRC cells (Fig. 4C, D; *p* < *0.05*). The results demonstrate that PARP1 may make a significant impact in CRC cells proliferation.

### PARP1 interacted with XRCC2 during SW620 cell proliferation

**Fig. 5:**
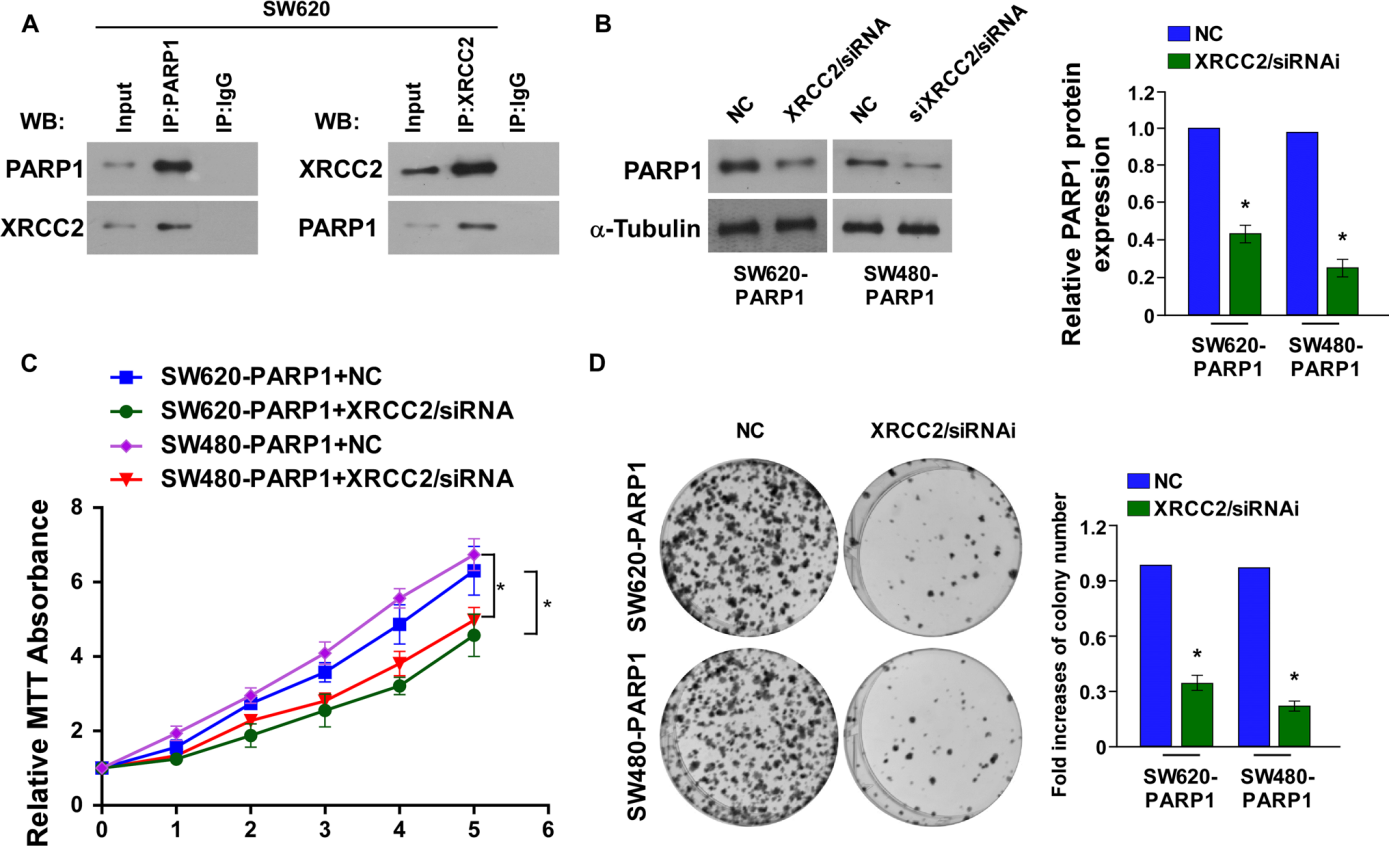
PARP1 interacted with XRCC2 regulates CRC cell proliferation. **A** The IP results of XRCC2 protein interacting with PARP1 protein. Input indicates the positive control group, IgG indicates the negative control group, IP indicates the target experimental group. **B** Western blot analysis of PARP1 expression. **C** CCK-8 assay and **D** colony formation assay to test cell proliferation of the PARP1 overexpression cells in SW480/SW620 transfected with NC, XRCC2/siRNA. Blot membranes were cut prior to hybridisation with antibodies during blotting and developed with X-ray film. *p < 0.05, (Student’s t test)

In previous researches, we found that the polymorphism or low expression of XRCC2 affected the sensitivity of CRC cells to PARP1 inhibitors [15, 18]. Based on previous theory, we assumed that PARP1 binds to XRCC2 in SW620 cells. To define the correlation between PARP1 and XRCC2, IP was performed. Using PARP1 and XRCC2 primary antibodies to perform two-way verification, both of which showed that, during SW620 cell differentiation, PARP1 interacted with XRCC2 (Fig. 5A). Western blotting result show that PARP1 expression was down-regulated while PARP1overexpression cells with XRCC2 inhibitor (Fig. 5B). Moreover, the cell proliferation was also inhibited which analyzed by CCK-8 assay and colony formation assay (Fig. 5C, D, p < 0.05). The result show PARP1 interacted with XRCC2 regulates CRC cell proliferation.

## Discussion

These traditional views believe that PARP1 had biological functions. For example, DNA repair, synthetic lethality, apoptosis, necrosis, histone binding, and so on [19]. After activated by DNA damage, PARP1 startup the DNA damage repair process as a tumor suppressor gene, binds to target proteins, DNA signal- and double-strand breaks, and other proteins of DNA repair [20]. However, the latest research showed that PARP1 participated nucleus to mitochondria communication, which involved in cell energy metabolism. Activation of PARP1 sparks off cell death and energy crumble, which indicate novel understanding on the significance of PARP1 activation. Therefore, the role of PARP1 in cells is undefined. Current researches have pointed out that PARP1 may be related to the CRC occurrence. Studies have proved that PARP1 (Ala762Val, rs1136410) was associated with the susceptibility to colorectal carcinoma of adult and children [21, 22]. Moreover, the study of Sakthianandeswaren has found that MACROD2 deletions or haploinsufficient caused chromosome instability (CIN) and impaired PARP1 activity in CRC. In turn, it drove the evolution of cancer [23, 24]. Therefore, we focus on the significance of PARP1 in colorectal cancer.

By Immunohistochemistry, real-time PCR, and western blotting, compared with normal tissues, we discovered that PARP1 was upregulated significantly in tumors. These lead us to further study the significance of PARP1 in the development of CRC. Furthermore, after analyzing the correlation between PARP1 expression and the CRC clinicopathological features, we surprised to find that the expression status of PARP1 was remarkably correlated with the degree of differentiation, TNM stage, distant metastasis, and depth of invasion in primary CRC. Increasing PARP1 expression may promote invasive behavior and metastatic process of CRC. However, the mechanism of how PARP1 affects the progression of CRC remains unknown. Previous researchers believed that PARP1 plays a crucial role in promoting the growth and proliferation of tumor cells. Santos [25] revealed that the expression of PARP1 is related to tumor location (tumor of colon or rectum) and tumor stage (III/IV or I/II grade). Similarly, Li [21] proved that PARP1 (Ala762Val) was associated with the susceptibility to CRC. Thus, it may not be surprising that PARP1 is involved in the metastasis and invasion of CRC. It worth further studies to clarify the mechanism involved.

What’s more, in this research we found that the positive PARP1 expression and CRC patients’ poor survival after surgery was correlated significantly. Patients with positive PARP1 expression had shorter OS and RFS compared with those patients with negative PARP1 expression. The two survival curves were remarkably different. The finding indicated that the PARP1 protein may affect the prognosis of CRC patients. However, the study of Li [21] found that the mutation of PARP1 (Ala762Val) may have nothing to do with the prognosis of CRC patients intrinsically. Therefore, to define the relevance between PARP1 and CRC patients prognosis, expand the sample and enroll more advanced CRC patients is necessary.

To further figure out the effect of PARP1 in CRC progression and development, cell lines stably expressing high (SW480-PARP1, SW620-PARP1) or low PARP1 (SW480-PARP1/RNAi, SW620-PARP1/RNAi) were constructed. Firstly, we investigated the affection of high or low PARP1 expression on the growth of SW480/SW620 cells. One of the important characteristics of cancer cell phenotype is unrestricted growth. The current result revealed that suppressing PARP1 effectively decrease proliferation, whereas upregulating it significantly promote proliferation, indicating that PARP1 could be an important regulatory factor for CRC cell proliferation.

Schaaf [26] found that PARP1 plays a vital part in the chemosensitization mechanism of hyperthermia of CRC. Previous study have shown that simultaneous deletion of two genes can lead to lethality in biological systems, otherwise the absence of one gene will be abided [25]. According to this concept, restraining PARP1 could be a latent therapeutic schedule for the therapy of cancers with defects in precise DNA repair genes, such as XRCC2, MRE11, and BRCA1/2 [26, 27]. A number of clinical trials have been launched with PARP inhibitors apply to CRC patients [27]. Furthermore, our study reveals that olaparib inhibits CRC cell proliferation in a dose and time-dependent manner [15]. These studies show that PARP1 expression can promote the proliferation of CRC cells. Moreover, PARP1 inhibitors have been shown to play an anti-tumor role in many tumors [15, 28]. Both PARP1 and XRCC2 related the double-stranded DNA HRR pathway. In the previous study, we found that CRC cell proliferation restrained by PARP1 inhibitor (olaparib) in a dosage and time dependent way. These findings indicated that there is a closed relationship between XRCC2 and PARP1. The effect of PARP1 inhibitor may require the presence of XRCC2. In the present study, we found that PARP1 interacted with XRCC2 in SW480/SW620 cells. However, up to now, there is no convincing evidence to define the correlation between XRCC2 and PARP1. In future experiments, we will further explore the close relationship between PARP1 and XRCC2.

In conclusion, we found that PARP1 was upregulated in CRC and promotes colorectal cancer cells proliferation. Furthermore, the expression of PARP1 in primary CRC was significantly correlated with the degree of tumor differentiation, distant metastasis, TNM stage, invasion, and survival. Lastly, the results of IP show that XRCC2 protein interacting with PARP1, the inhibitor of XRCC2 can reduce the proliferation arousing by upregulation of PARP1, but need further explore.

Several limitations that need to be clarified. Firstly, this was a retrospective study, which inevitably has selection bias. A multicenter with a bigger sample size would be more powerful to draw such a conclusion. Secondly, it should be verified again in other different colorectal cancer cell lines both in vitro experiments and in vivo experiments to further demonstrate the ability of high expression of PARP1 levels play a vital role in CRC. The findings should be validated in more physiologically relevant models. Last but not least, further research will be essential to fully understand the clinical implications and therapeutic potential of targeting this interaction in the management of colorectal cancer.

## Data Availability

The datasets used and analyzed during the current study are available from the corresponding author upon reasonable request.
